# An Exploration of Child–Staff Interactions That Promote Physical Activity in Pre-School

**DOI:** 10.3389/fpubh.2021.607012

**Published:** 2021-08-02

**Authors:** Karin Oddbjørg Kippe, Tom Stian Fossdal, Pål Arild Lagestad

**Affiliations:** Department of Teacher Education, Nord University, Levanger, Norway

**Keywords:** kindergarten, children, pre-school staff, physical activity, health

## Abstract

A previous study identified a significant association between the average physical activity levels of pre-school staff and children during pre-school hours but did not determine if this association was initiated by pre-school staff or children. The present study aimed to explore the interactions between children and staff to better understand the conditions of such a relationship. Observations using the Environment and Policy Assessment and Observation (EPAO) protocol were carried out in three pre-schools, and a focus-group interview and semiformal interviews were conducted with five pre-school staff in one of the three pre-schools to examine the research question. Both the observations and the interview data revealed that physical activity by pre-school staff initiated physical activity among the children. The findings pointing to the importance of pre-school staff as organizers of the physical activity of children and the willingness of staff to join a physical activity initiated by the children also appear to be important. The findings also indicate that most of the physical activity took place outdoors. The study highlights the importance of pre-school staff as major contributors to the physical activity of pre-school children.

## Introduction

Lifestyles characterized by obesity and physical inactivity exhibit a tendency to persist from early childhood to adulthood (1–3). Although Norwegian studies about the physical activity levels of pre-schoolers have revealed a fairly high percentage of children meeting physical activity (PA) guidelines (4–6), several international studies have shown that pre-schoolers are not as active as initially assumed (7–10) and point to the time that children spend indoors as constituting a limiting factor (11). Longitudinal studies report that sedentary time already begins to increase from the ages of three to five (12). Moreover, a cross-sectional investigation conducted by Cooper et al. (13) found that the total amount of physical activity decreases by an average of 4.2% each year from the age of 5 to the age of 18. Bearing this in mind, Goldfield et al. (14) claim that physical activity should be prompted as early as possible since the activity patterns of children are more easily influenced by the attitudes of role models. In addition, the foundation for a physically active lifestyle is formed by bodily experiences as young children (15, 16), and thus, children should be introduced to the paradigm that physical activity is very enjoyable (17). The Norwegian pre-school framework plan points out that physical activity promotes positive attitudes and actions that are considered crucial for the perception of physical activity among children (18). This can be achieved through the perception of mastery in physical activity among children. According to both Rossem et al. (1) and Borraccino et al. (19), lifestyle behavior will follow the same trend from pre-school age into adulthood. According to this point of view, physical activity levels in pre-school can affect health both in the short term and in the long term. Finn et al. (20) found that more than 50% of the average daily activity occurred during pre-school hours of children, while Fossdal et al. (21) found that Norwegian pre-school children had 66% of their average daily activity during pre-school hours. Nilsen et al. (4) found that children had 77% of their daily physical activity in pre-school. This demonstrates that pre-schools serve as an important arena to reach as many children as possible, especially given that 97% of Norwegian children aged from three to five attend a pre-school (22). Research also points out that pre-school is a setting in which children can meet and interact with pre-school staff with high physical activity levels (6, 21). Several studies have found positive effects of activities structured by pre-school staff in pre-schools (11, 23–26). An investigation also identified positive associations between the objectively measured activity level of pre-school children (aged 4–6) and pre-school staff (21). Bower et al. (27) found that pre-schoolers are more likely to reach a higher activity level during activities initiated by pre-school staff who participate in, and have positive attitudes on, physical activity. Adult involvement in play situations and physical activity might, for instance, lead to more recognition for children. This was found to especially be the case through interaction and collaboration (28), where it has been found essential that staff promote physical activity and a healthy lifestyle (29). Moreover, several studies claim that engagement in terms of encouragement, praise, and recognition may affect the activity levels of children in a positive direction (11, 30, 31). The individual attitudes and behavior of pre-school staff may therefore play a key role in promoting the physical activity of children (29). While playing, children develop the gross motor skills necessary for physical activity.

It has been shown that the physical activity levels of pre-school staff and pre-school children are interrelated (6). The present study aimed to explore the interactions between children and staff to better understand the conditions of such a relationship during the pre-school time in middle-active pre-schools. Moreover, this study also aimed to explore the role of pre-school staff as the initial driving force for the physical activity of children in pre-school. In this study, physical activity is defined as a gross motor activity. Furthermore, child–staff interactions are defined as interactions that affect physical activity.

## Materials and Methods

In the present study, observations of pre-school staff in interaction with pre-school children, a focus-group interview and semiformal interviews of pre-school staff, have been used to explore the relation between the physical activity of pre-school staff and children during pre-school hours.

### Subjects and Procedures

Observation data were collected in three middle-active pre-schools during a 3-day observation in October 2017 in one pre-school and during a 2-day observation in April 2018 in two other pre-schools. The present study elaborates on a previous investigation in 13 Norwegian pre-schools of the physical activity level of the 4-to-6-year-old children, measured using an accelerometer (6). A one-way ANOVA revealed that moderate to vigorous physical activity (MVPA) of pre-schoolers in pre-school is significantly different between the 13 pre-schools where pre-schools 12 and 13 had significantly higher activity levels than pre-schools 1–4. Pre-school 12 and 13 is defined as high active, pre-school 5–11 is defined as middle active, and pre-school 1–4 as low active. In the present study, three of the middle-active pre-schools were selected for participation using stratified selection.

Table 1 shows the pre-schools categorized according to average physical activity level among pre-schoolers in the low-, middle-, and high-physical active pre-schools.

**Table 1:** Pre-schools categorized according to average physical activity level among pre-schoolers into low-, middle-, and high-physical active pre-schools.

**Pre-school**	**Mean MVPA**	**SD**	***N***
Low activity pre-school	50.1	11.9	74
Middle activity pre-school	59.3	17.2	136
High activity pre-school	73.1	22.5	34

The children in the middle-active pre-schools had an average of 59.3 min of moderate to vigorous physical activity per day. A focus-group interview (32) has been carried out with five of the six staff members in one middle-active pre-school. The focus-group interview has been used to help the research participants remember different incidents or to elaborate on descriptions of incidents or experiences the group members have in common (33). In the spirit of transparency and to ensure the credibility of the findings, information about the focus-group participants, data collection, and data analysis are given below.

All of the informants worked full-time, mainly with children between 4 and 6 years of age. The focus-group interview took place in April 2018. A condition for participation in this interview was that the participants had answered an individual questionnaire about their personal activity habits and their views on physical activity in the study of Kippe and Lagestad (6). The focus interviews were conducted at the pre-schools in a room with only the researcher and research participants in attendance. The interviews were conducted after the closing time of the pre-school. The participants talked freely and also much with each other. As a researcher and the moderator, the researcher assumed a background role. This allows the participants to present their opinions, and all were encouraged to speak. By acting as a moderator, the researcher can ask general questions about the themes to encourage participants to communicate thoughts (34). This is done through the topics in the interview guide having sub-questions that could help the staff. The focus-group interview was structured, using an open semiformal interview guide, using some structured questions related to the three overarching themes: (1) “The pre-school staff” with sub-questions about which factors they believe create physical activity, their role in physical activity of children, and the degree to which they encourage physical play among children, initiate physical play with children, and participate in physical play with children; (2) “organization of physical activity” with sub-questions about the degree to which they plan and organize physical activity, which type of physical activities they plan, duration of activities, and whether the activities take place outside or inside; and (3) “physical environment” with sub-questions about the size of the area outside and inside, in which areas the children prefer to play, and which other areas outside the pre-school they use for physical play. These themes were also used in the open semiformal interviews that were conducted with pre-school staff in pre-school 1 during the observations.

The pre-school staff received written and oral information about the procedures and ethical standards prior to signing the written consent form. They were also informed that the study was voluntary and had been approved by the Norwegian Social Science Data Services (NSD). The parents of the children who were observed during the study have given written consent for the participation of their child in the study.

To explore the relation between the physical activity of pre-school staff and children, three pre-schools were observed by a different observer in each pre-school. The observation took place among pre-school staff who worked mainly with children of 4–6 years old, and during the time period that was categorized as pre-school hours (08.00 a.m. to 15.30 p.m.) of children and staff. This is in line with the Environment and Policy Assessment and Observation (EPAO) protocol, which consists of a day-long observation (27).

An initial observation has been used as the basis for exploring the relation between the children and pre-school staff in physical activity in the interviews. The EPAO protocol is a tool developed when using direct observation to evaluate the physical activity environment, policies, and practices in pre-school. One strength related to the use of the EPAO protocol is the use of direct observation, which is a more valid goal compared to self-reporting (30). Furthermore, direct observation is appropriate when assessing complex environments (35). According to Bower et al. (27), a key finding was that pre-schools with higher physical activity environment scores had children who were more physically active and less inactive while in child care. For this reason, an initial observation was used with the registration of the number of child-staff interactions as a starting point for obtaining in-depth information in interviews. The relationship between the physical activity of pre-school staff and children was directly observed, while the observer was present with children and staff. The EPAO protocol was developed to quantify both social and physical environmental factors thought to affect dietary and physical activity behavior. The physical activity portion of the EPAO consists of eight subscales, where we investigated the subscale “Staff behavior and active opportunities” (27). This method is similar to the EPAO instrument used in other observational studies relating to the physical activity of pre-schoolers (27, 36) but has been simplified due to the narrowed focus area in the present study.

### Analyses of Observation Data

Due to the investigation of only two subscales in the EPAO protocol, we did not assign scores to each item, but rather we observed and measured gross motor activity (walk, run, jump, crawl, throw and grab, roll, balance) in a tally in the subscale “Staff behavior” according to three themes: (1) initiation of physical activities by pre-school staff among children, in which pre-school teachers participated with the children, (2) initiation of physical activities by pre-school staff among children without participating themselves, and (3) participation when pre-school staff spontaneously joined child-initiated physical activities. Within each theme in the subscales “Staff behavior,” the number of times pre-school staff initiated physical activity by inviting the child into physically active play, where the pre-school staff participated themselves, how often they invited children into physical activity without participating, and how often pre-school staff participated in physical activity when invited by the children were recorded. For the subscale “Active opportunities,” the items indoor and outdoor were investigated, where the frequency of items in staff behavior was registered both indoors and outdoors. However, a criterion in the measurement of the three themes in the behavior of the staff was a minimum of 1.5 min of engagement in physical activity (37). An activity could involve one, several, or many children and could be planned, spontaneous, or part of a daily routine. Another criterion was to first select the most obvious and distinct activity during the observations (37).

### Analyses of Interview Data

The data from the focus-group interview and the semiformal interviews were transcribed and analyzed using QSR NVivo 10 software. The analyses were based on transcribed answers and focused on meanings, as described by Johannessen et al. (38). Reading the text led to the formulation of themes from the statements of pre-school staff. Such an approach is preferred when researchers intend to describe and understand common interpretations of a certain phenomenon by pre-school staff (33). With such a strategy, opinions and statements in relation to the link between the activity levels of pre-school teachers and children are identified as themes. The data material has been processed in three steps in accordance with Kvale and Brinkmann (39).

#### Step 1: Self-Understanding

First, an impression of the text through the transcriptions of the focus-group interview and the semiformal interviews was formed. The analysis commenced with a careful reading of the transcribed interviews to gain an impression of the content. During transcription, the emerging reflections and ideas were noted. Thus, thoughts about the social and emotional aspects of the interview situation can be captured at an early stage (39). The noted reflections and ideas were preliminary, based on the excerpts from the interviews, and were anchored in the data. Furthermore, the first step compacted the text into meaning units (39). The immediate meaning has then been compressed into shorter sentences. An example may illustrate how the meaning units are anchored in the data:

*I think it's the adults who make the children active. What we adults do is reflected in the children. After all, some children need an adult to join. I have to be a role model*.

*It's good the more things they can do. That we go climbing or skiing, or for a walk in the hills or go on regular trips, walk along the beach, facilitated activity, that the area is organized. So we try to vary things. We have to go on trips if we're to make sure that everyone is physically active*.

This quote contains meaning units, for example, “make the children active,” “need an adult to join,” “the area is organized,” and “try to vary.”

#### Step 2: General Interpretation

In the second step, the meaning units that emerged in Step 1 were given theoretical labels. The aim of using keywords was to lift the descriptions to a theoretical level (39). For example, “adult role” may be the theoretical label for the need for an adult to join. In this case, a theoretical label emerged from, for example, this excerpt:

*After all, some children need an adult to join*.

The essence of the keywords and short sentences was revealed and expressed in transformed theoretical labels. The various details and shades of the keywords relating to the adult role were given the same theoretical label with the aim of discovering what remained consistent throughout the variations.

*They see that we're happy, and skip and jump, then they will too*.

For the excerpt above, adults may be considered as role models, participants in physical activity: we're happy. The variations are all about the adult role. The skipping and jumping of adults expressed a willingness to create possibilities for physical activity. Role model is then a theoretical label. Furthermore, the adult may also be considered as an initiator and a motivator, which refers to both the organization of physical activity and the adult role. The meaning units are transformed to theoretical labels relating to the adult role and initiation of physical activity.

#### Step 3: Theoretical Understanding

In the third step, theoretical labels were clustered into three major themes. The statements of pre-school staff were sorted and clustered into these three themes. As a result of the analysis process, based on excerpts from the interviews, the material has been interpreted and analyzed within the themes “the pre-school staff,” “organization of physical activity,” and “physical environment.” The themes are the same as the overall themes in the interview protocol. The questions in the protocol are based on previous research on conditions that create and increase physical activity in pre-school. The analysis process is presented in Table 2.

**Table 2:** Examples from the analysis of the interviews.

**Excerpts from interviews**	**Meaning unit**	**Notes and reflections**	**Theoretical label**	**Themes**
I think it's the adults who make the children physical active. What we adults do is reflected in the children. After all, some children need an adult to join. I have to be a role model.	The adults who make the children active Need adults to join Role model	Encourage Motivator Children's perspective	Facilitate Role model Organize	Pre-school staff
Can we do that? Can you join? They know that we mostly try to be with them.	Try to be with them	No rigid daily rhythm Adults participate	Adult Participant	Pre-school staff
They see that we are happy, and skip and jump, then they will too.	Skip and jump	Willing to be physically active	Role model	Pre-school staff
It's good the more things they can do. That we go climbing or skiing, or for a walk in the hills or go on regular trips, walk along the beach, facilitated activity, that the area is organized. So we try to vary things. We have to go on trips if we're to make sure everyone is physically active.	Go climbing, skiing, for a walk in the hills, or go on regular trips	Organize Flexibility Variety Facilitate Give everyone the opportunity to be active	Variety in use of the area's affordances Organizing and flexibility	Pre-school staff

Various interpretation and perspective alternatives were discussed among the three researchers. This contributed to intersubjective consensus in the analysis, thus further strengthening the credibility of the findings.

## Results

Descriptive data referring to the pre-school staff and children in the three pre-schools are presented in Table 3.

**Table 3:** Descriptive data referring to pre-school staff and children in three pre-schools.

**Pre-school**	**Pre-school staff (*N*)**	**Pre-school children (*N*)**	**Pre-school staff, age**	**Trained pre-school teachers (%)**
Number one	6	19	41.3	33.3
Number two	3	16	37.7	66.6
Number three	3	17	47.3	66.6

The findings from the analysis of both the observations and interview data revealed that instigation of physical activity in pre-schools is complex and initiated by both the pre-school staff and the children. However, both the observations and the interview data revealed that children liked it when the adults participated in physically active play. The pre-school staff claim that the adults make the children physically active.

The first main finding was the importance of pre-school staff as organizers of physical activity of children. This was especially the case with the focus of pre-school staff on making inactive children active by encouraging physical activity and sometimes taking the initiative to engage in physical activity with them. This was evident from the analysis of the observations and interviews. The interviews revealed that some children need an adult to join. Some of the staff gave the children advice on how to get back into the game or on what kind of game they could try (e.g., role-playing a police officer capturing thieves on bikes), while others joined the children to initiate a new game. In another example, one pre-school arranged a day of activities in the snow. In these activities, most children preferred to be active using sleds to slide down an incline. The pre-school staff gave the children sleds to slide on, observed the activity, and helped them. However, the pre-school staff also initiated other activities with the children, such as digging snow holes and skiing. In another pre-school, the staff arranged a mile-long ski tour with different games along the way that required equipment to be set up by the staff. This is registered under pre-school 1 as initiated without participating in Table 4. Moreover, during the interviews, when a pre-school staff member was asked who initiated physical activity in the pre-school, she pointed to herself as the main initiator:

Sometimes the pre-school staff, sometimes the children, but mostly the pre-school staff. Obviously, if we don't initiate physical activity and take the children with us and are physically active together with them, there will not be that much physical activity.

**Table 4:** The number of times pre-school staff initiate and/or participate in physical activities with the children, indoors, and outdoors, during the 3-day observation.

	**Indoors**	**Outdoors**
**Pre-school 1**		
Initiate (participating)	7	11
Initiate (without participating)	0	8
Participation (without initiating)	2	14
**Pre-school 2**		
Initiate (participating)	4	14
Initiate (without participating)	4	10
Participation (without initiating)	4	8
**Pre-school 3**		
Initiate (participating)	2	6
Initiate (without participating)	5	6
Participation (without initiating)	3	7

Pre-school staff pointed to their key role as organizers: “Our mission is to organize so it is easy for children to become physically active, or to initiate an activity if that is necessary. In that way, I think we serve as a model for the children.” On one occasion, the pre-school staff took a wireless speaker out in the snow and started to dance. This is registered under pre-school 1 as initiated and participating in Table 4. The children immediately began to dance together with them. During the interviews, the pre-school staff were asked if the children would have started to dance without their initiative: “No, I don't think so. They look at us and our mood when we're dancing. They see the dancing is making us happy, that we skip and jump, so they also want to do it. Really, I believe so.” The results from the interviews also point to the importance of pre-school staff in a longer perspective: “I think we offer a lot of ideas [according to physical activity]. However, it's not certain that we see they are Doing these things the same day.” The pre-school staff state that, in many of the physical activities that they do, such as climbing, skiing, and playing, the children engage in role-playing the following week perhaps or even later. In other words, according to several of the pre-school staff members, activities introduced in pre-school will also create physical activity years later: “That they get some experiences with skiing and other activities, and think,” “Oh, that's an activity I want to do again.”

The second main finding was the importance of the willingness of pre-school staff to join physical activity initiated by the children—a strategy that both the interviews and observations showed was crucial for the physical activity levels of children. During the observations, we discovered that many children tried to involve the pre-school staff in activities that demanded physical activity, and the response of the pre-school staff could both escalate the activity (by joining the children) or deflate it (by not joining the children). Table 4 shows that, in pre-school 1 and 3, the staff more often participate in the physical activity initiated by the children than initiated by the staff themselves. During the interviews, this finding was also evident, exemplified by the statement: “I do feel that the activity level among the children around you increases when I participate a little bit in the play.” The pre-school staff also stated that many children come to them and ask such questions as, “Can we do that?” and “Can you please come along?”, and highlight the importance of joining the activity with the children. One staff member supported this notion when she said that “As an example, when they ask twice and don't get a concrete answer. Then, they won't ask a third time,” and pointed to the fact that children find it somewhat exciting when the pre-school staff join the activity. From the stories by pre-school staff, the message is that the children would have been less active if the pre-school staff did not join the physical activities when the children asked them. A staff member explains: “If we had said, ‘not now,’ he would just go and sit down in the sandbox. But, when we say, ‘Yes, we'll join,’ then the activity actually takes place.” In recollecting about the children sliding on their sleds down an incline, a pre-school staff member stated:

If every kid had run up and down, then we appropriately would not have said “Can we join? Can we join?” But, the kids want us to join them, and some kids really need us to join [to continue the activity]. There are some kids who will not join the activity unless the pre-school staff join in. If not, they'll lie up on the hill…[…]. Because some kids find it very strenuous to go up that hill, and then I have to participate as a role model.

Another observational finding was that, when other children noticed that one of the pre-school staff was playing with a child, many wanted to join the new game. On one occasion, a pre-school staff member initiated a “catch and run” game outdoors and many children immediately came to join this game. This suggests that children enjoy it when the pre-school staff participate in games and are likely to join games when the staff are involved. Moreover, some of the children seemed to be very physically active by nature, but, for those who were not, games initiated by pre-school staff often involved a more physical game than what they normally have taken part in. However, when pre-school staff were involved in the game, children seemed to spend more time in activities that involved more physical play. We also observed the importance of participation by pre-school staff during skiing, where the children who participated together with the staff who also were skiing were much more active than children skiing on their own. During the interviews, pre-school staff stated, “Children seldom find out that they want to play tag all by themselves. But, if we participate, the activity lasts much longer.” Furthermore, “If we're active, participate in the activities and play soccer with the children, the speed of the activity also increases among them.”

The third main finding was that, even if the pre-school staff played important roles as contributors and participants in the physical activity of children, most of the children were quite active outdoors and played by themselves. Moreover, the pre-school staff often took on the role of observer, and due to the varied activity levels amongst the children, it was obvious that it was children playing that was important to the pre-school staff. The observational data also showed that, mostly, the children engaged in “free play” indoors. The observation revealed that less physical activity is organized indoors than outdoors in all pre-schools in the present study (see Table 4). However, in the focus-group interview, two pre-school staff members said, when presented with the findings, “If the kids have fun in role play and play together, then I think it's more important than getting the kids running.” These statements were confirmed by several other statements, such as, “On days that we are on trips, we take the lead. But when we are here [in pre-school], the kids are the ones who mostly take the lead.” However, it is worth noting that pre-school staff played a key role in organizing the activities of children by providing them with the objects they needed for play and assisting them in various ways.

The fourth main finding was that indoor activities were characterized as being mostly sedentary, and children were not allowed to run or shout indoors unless pre-school staff were involved, which may be due to several factors, including limited space indoors. This finding is specified in Table 4.

However, in this setting, pre-school staff took on a new role and used most of their time indoors sitting next to the children and participating in sedentary activities, for instance, playing with Lego, solving puzzles, drawing, cooking, or performing other duties. There were also rules specifying that running, jumping, climbing, and other high-intensity activities were prohibited indoors. This finding was also confirmed by interview data when a pre-school staff member was asked about indoor and outdoor physical activity at pre-school:

Indoors, they should be quiet. After we have been outdoors, they have to practice to be calm indoors. Some of the children really need it! Should they be allowed to be wild indoors? Why? It's really about learning good manners. You know, they'll soon be starting in primary school, where they are expected to only walk indoors. So, the earlier they learn this…

As shown in Table 4, pre-school staff also initiated physical activities indoors, but these activities were always organized and controlled by one of the pre-school staff. These organized physical activities could last from 15 to 30 min, and the intensity seemed to vary. Moreover, organized physical activities indoors seemed to be planned, which was not always the case outdoors, where pre-school staff often initiated games spontaneously. Moreover, observations indicated that the pre-school staff only encouraged physical activities when a member of the staff participated in the activity when they were indoors. This seemed to be contradictory to what most of the pre-school staff did outdoors when children dropped out of the games. This also suggests that there are types of rules or expectations relating to what both the children and the pre-school staff are supposed to do in various situations, as indicated by the above excerpt from the interviews. Similarly, the pre-school staff joined children in outdoor physical activity more frequently than indoor physical activity (see Table 4). However, the observations indicated that the involvement of pre-school staff in the physical activity of children, whether or not it was initiated by the pre-school staff, has an effect on the physical activity of children, as they tend to become more eager when pre-school staff play along on the terms of children. Overall, both the observations and interview data revealed that pre-school staff had a larger effect on the activity levels of children than the other way around.

## Discussion

This study indicates that pre-school staff have the power to impact the physical activity of children when they initiate and participate in the physical activities of children.

The first main finding was the importance of pre-school staff as organizers of the physical activity of children, and their influence on the participation in physically active play of children. This is in line with the findings of Mikkelsen (29), showing that encouraging play and movement is associated with more children undertaking moderate activity. Brown et al. (11) also found that the initiators of activities in pre-school were pre-school staff, with 81% of activities being initiated by them. On the other hand, Kallestad and Ødegård (37) found that most of the activities in pre-school were a result of initiatives of children. However, they only studied the spontaneous activity and not organized activity. As pointed out, the present study indicates that the physical activity of children is associated with the organization of physical activity among children by the staff. Intervention studies (23, 31, 40, 41) have also reported that activities structured by pre-school staff might increase the daily physical activity levels of children. Nevertheless, the study of Bjørgen and Svendsen (42) demonstrated that pre-school staff emphasized the role of being a facilitator and supporter. This points to the importance of prioritizing enough time to observe the children and make it possible to intervene and assist the less physically active children so that they can become more active. The interviews also revealed that, when the pre-school staff initiated activities, the children spent more time engaged in them. Furthermore, when games of a more physical nature were played, the physical activity of the children, who were normally not as active, was encouraged to participate. This corresponds to findings by Bower et al. (27) who showed that pre-schoolers are more likely to reach a higher activity level during activities initiated by pre-school staff, who participate and express positive attitudes about the physical activity. Tonge et al. (43) highlight the positive relationship between the sedentary behaviors of the educator and children and underline that improving physical activity and sedentary behaviors of educators will likely improve the physical activity levels of children. Pre-school staff who participate in children games might provide children with new ideas that precede the game and actively support the mastery of children through involvement and reflection.

There is a general agreement among several researchers (11, 17, 28, 30, 44) that positive adult encouragement is especially important when pre-school staff participate in the physical activities of children. Initiation of physical activities of pre-school staff or participation and involvement in children-initiated games that are considered to be physical may therefore play a key role in the daily physical activity of children. Bjørgen and Svendsen (42) also point out that another reason for pre-school staff wanting to participate in the physical activity of children is because being involved in their activities is connected with feelings of being meaningful to the children. This is in line with the present study, which finds that some children seemed to be very physically active by nature. For the children who were less physically active, the pre-school staff initiated games that required physical activity.

The second main finding was that the willingness of pre-school staff to join the physical activity initiated by the children was of major importance for the physical activity of the children. The observations indicated that, even if the pre-school staff played important roles as contributors to and participants in the physical activity of children, most of the children were quite active outdoors and played by themselves. This is in line with Kallestad and Ødegård (37), who reported that ~80% of the activities in pre-school were unplanned, and these activities were the result of the initiatives of children. It was also found in that study that pre-school staff often have a passive role. Moreover, Brown et al. (11) reported that teacher-arranged physical activities were found in only 2.6% of the observations. Hagen (45) showed that pre-school staff encourage children to play by themselves. In that study, the children were expected to be more independent and physically active outdoors, although our observations indicate that children derive great enjoyment from pre-school staff playing along with them as equals. However, research also indicates that not all pre-school teachers believe that the involvement of pre-school staff is the most important factor in children games and physical activity. Cashmore and Jones (46) reported that the pre-school staff in their interview study considered child-directed play as the most valuable factor and were therefore reluctant to interfere. Furthermore, pre-schools are obligated to provide conducive conditions for play, friendship, and the culture of children (18). This might explain in part why child-directed play is so prominent in pre-schools and emphasizes the importance of socialization that occurs between children. Nonetheless, Copeland et al. (47) showed that the pre-school staff in their interview study claimed that they held the key to the physical activity of children, as they were the ones to decide which opportunities children should have to be physically active, in addition to the degree of involvement or dedication they should have with the children. Moreover, several researchers have identified portable equipment and toys as a key factor for physical games of children (11, 27, 30, 48, 49), indicating that pre-school staff do not have to interfere as long as children have opportunities to play while they are in motion. Furthermore, Brown et al. (11) showed that MVPA was recorded during 19.5% of the intervals that were child-initiated, whereas MVPA was coded for 15.4% of the outdoor activities initiated by the pre-school staff. Although the observations of the present study reveal that children play well on their own, pre-school staff need to be aware of the individual games the child so that children who are not as naturally active are helped to be sufficiently physically active during pre-school hours. Furthermore, a new study using accelerometery among Norwegian pre-school staff found that, in general, they had a high activity level during work (50). The same study showed that those who work with older children (4–6 years old) had the highest activity levels with 55 min of MVPA per day, which is more than other Norwegian women (34.3 min of MVPA per day) and men (36.5 min MVPA per day) in the same age group as the pre-school staff in the present study (51).

The third main finding was that, even if the pre-school staff played important roles in the physical activity of the children, most of the children were quite active outdoors and played by themselves. Kallestad and Ødegaard (37) pose an interesting question about whether or not all children will be included in physically active play if they initiate the activity themselves. The study of Kippe and Lagestad (6) shows that there is a positive association between MVPA during leisure time and MVPA in pre-school, in which MVPA in pre-school increases when MVPA during leisure time increases. In general, pre-school increases such differences and contributes to creating even greater differences between low-active and high-active children (21). O'Neill et al. (52) found that children who did not meet PA guidelines in school did not “catch up” with children who met the guidelines. This underlines the importance of increasing the level of physical activity for all children in pre-school and may imply that pre-school staff must initiate, organize, and perhaps participate in physically active play to a greater extent than they currently do (53).

The fourth main finding was that a distinct difference in physically active play was found between the time that children spent indoors and outdoors during pre-school hours, and indoor activities were characterized as more sedentary than outdoor activities. The observations suggest that pre-school staff have an impact on the physical activity of children based on actions and probably mediated expectations, which might explain, in part, the association between pre-school staff and the physical activity of children. The findings indicate that pre-school staff enter situation-dependent roles through which they might demonstrate to the children that indoor activities should be less physically active and with less noise than outdoor activities. The fact that all of the physical activities indoors had the involvement of the pre-school staff supports the idea of a controlling role for pre-school staff when indoors. For children, this might affect their amount of daily physical activity, which has also been identified by Brown et al. (11), who identified the time that children spend indoors as a limiting factor for their daily physical activity. As pre-school has been identified as a major determinant of the physical activity of children (20), pre-school staff are advised to take more responsibility for initiation of and participation in children games, both indoors and outdoors.

## Strengths and Limitations of the Study

The present study has several positive features. First, to the best of our knowledge, this is the first study to examine the interaction between the physical activity of pre-school staff and pre-school children. It is also the first to elucidate if this interaction is initiated mainly by pre-school staff or children and to examine the extent to which these factors influence the physical activity of children in pre-school. Another advantage is the mixed methods design with the use of interviews and observations, as they can complement each other, thus providing more complete and deeper information on whether the physical activity of children is initiated mainly by pre-school staff or the children themselves. Several intervention studies [for example (40, 41)] show that activities structured by pre-school staff might increase the daily physical activity levels of children.

However, the present study is not without limitations. One main limitation is that although the observer has taken an objective stand, all individuals have subjective perceptions of what is occurring. Moreover, the observations were conducted by a different observer in three different pre-schools. It would have been preferable to have several observers in each pre-school and additional pre-schools to increase the robustness and generalizability of the study. Observations should certainly also be made for a longer period and should be made in more schools. Only staff from one pre-school were interviewed; more pre-schools would have been preferable. Furthermore, the interviews reflect the subjective opinions of pre-school staff about the interaction between the physical activity of pre-school staff and pre-school children and whether or not this interaction is initiated mainly by pre-school staff or children. The interviews also reflect which factors influence the physical activity of children in pre-school. Observations made in April and October may have affected the physical activity of children as research shows that children are more active in the summer than in the winter (4). In this study, however, this does not have an effect on our findings because we were looking for the interaction between children and staff. Although the present study identified some factors that influence the physical activity of children in pre-school, other research designs with more data may have identified more factors. If more quantitative data were collected through systematic observation of the activities of children (types, intensity, and duration, or the total duration of staff-initiated and child-initiated activities), it could support our findings to a greater extent.

## Conclusion

The findings strongly identify pre-school staff as the main organizers of the physical activity of children in pre-school through their initiation of physical activity and their willingness to participate in physical activity when children initiate such activity. Moreover, the indoor physical activity of the pre-school seemed to be of minor importance. Both the observational and interview findings indicate that children enjoyed physical activity when pre-school staff participated and played along with them as equals, and in that way, the pre-school staff had an effect on the participation of children in physical activity. This demonstrates the importance of physically active pre-school staff, as they might have the power to substantially affect the physical activity of children based on actions and mediated expectations. Further research should use a longitudinal design to explain any side effects resulting from encouraging physical activity in terms of initiation, participation, and general attitudes toward physical activity of pre-school staff and primary guardians for children.

## Data Availability Statement

The raw data supporting the conclusions of this article will be made available by the authors, without undue reservation.

## Ethics Statement

The studies involving human participants were reviewed and approved by Norwegian Social Science Data Services (NSD). Written informed consent to participate in this study was provided by the participants' legal guardian/next of kin.

## Author Contributions

KK and PL contributed to the design and writing of the introduction, methods, discussion, and conclusion sections. TF contributed to the methods section. All authors contributed to the article and approved the submitted version.

## Conflict of Interest

The authors declare that the research was conducted in the absence of any commercial or financial relationships that could be construed as a potential conflict of interest.

## Publisher's Note

All claims expressed in this article are solely those of the authors and do not necessarily represent those of their affiliated organizations, or those of the publisher, the editors and the reviewers. Any product that may be evaluated in this article, or claim that may be made by its manufacturer, is not guaranteed or endorsed by the publisher.
